# Endoplasmic reticulum-quality control pathway and endoplasmic reticulum-associated degradation mechanism regulate the N-glycoproteins and N-glycan structures in the diatom *Phaeodactylum tricornutum*

**DOI:** 10.1186/s12934-022-01941-y

**Published:** 2022-10-20

**Authors:** Jichen Chen, Hong Du, Zidong Liu, Tangcheng Li, Hua Du, Wanna Wang, Muhammad Aslam, Weizhou Chen, Ping Li, Haodong Luo, Hao Fang, Xiaojuan Liu

**Affiliations:** Institute of Marine Sciences, Guangdong Provincial Key Laboratory of Marine Biotechnology and STU-UNIVPM Joint Algal Research Center, College of Sciences, Shantou University, Shantou, Guangdong China

**Keywords:** Tunicamycin, N-glycosylation modification, ER-quality control, ER-associated degradation, N-glycan, *Phaeodactylum tricornutum*

## Abstract

Tunicamycin inhibits the first step of protein N-glycosylation modification. However, the physiological, transcriptomic, and N-glycomic effects of tunicamycin on important marine diatom *Phaeodactylum tricornutum* are still unknown. In this study, comprehensive approaches were used to study the effects of tunicamycin stress. The results showed that cell growth and photosynthesis were significantly inhibited in *P. tricornutum* under the tunicamycin stress. The soluble protein content was significantly decreased, while the soluble sugar and neutral lipid were dramatically increased to orchestrate the balance of carbon and nitrogen metabolisms. The stress of 0.3 μg ml^−1^ tunicamycin resulted in the differential expression of ERQC and ERAD related genes. The upregulation of genes involved in ERQC pathway, the activation of anti-oxidases and the differential expression of genes related with ERAD mechanism might be important for maintaining homeostasis in cell. The identification of N-glycans, especially complex-type N-glycan structures enriched the N-glycan database of diatom *P. tricornutum* and provided important information for studying the function of N-glycosylation modification on proteins. As a whole, our study proposed working models of ERQC and ERAD will provide a solid foundation for further in-depth study of the related mechanism and the diatom expression system.

**Supplementary Information:**

The online version contains supplementary material available at 10.1186/s12934-022-01941-y.

## Introduction

*Phaeodactylum tricornutum* is a diatom that is used as a model organism for studying a number of biological processes such as carbon and nitrogen metabolisms, lipid biosynthesis, carbon cycle in ocean and photosynthesis [8]. Studies on protein glycosylation pathway, especially on the protein N-glycosylation pathway have received very little attention so far. The pathway of protein N-glycosylation modification was only recently proposed in *P. tricornutum* [14, 29]. In N-glycan processing, a Man5GlcNAc2-oligosaccharide intermediate was first synthesized and connected to a dolichol pyrophosphate on the cytosolic side of the endoplasmic reticulum (ER) [19]. After the action of flippase, the N-glycan was transferred into the lumen of ER and continued to synthesize the N-glycan precursor Glc3Man9GlcNAc2-PP-dolichol. Subsequently, the precursor was transferred from the PP-dolichol to the asparagine residue of a nascent protein by the oligosaccharyltransferase (OST). The N-glycosylated protein was then deglucosylated and/or reglucosylated by a series of enzymes for the quality control. These enzymes include α-glucosidase I/II, calreticulin (CRT), UDP-glucose: glycoprotein glucosyltransferase (UGGT) and α-mannosidase I. On this step, CRT was an important ER chaperone protein for the ER quality control (ERQC) (Nagashima et al. 2018a). Correctly folded and modified N-glycoproteins will be exported from ER to the Golgi apparatus for further N-glycosylation modifications. Finally, different species specific N-glycans were synthesized in the Golgi apparatus, including high mannose-type, paucimannosidic-type, hybrid-type and complex-type N-glycans [4, 14]. However, incorrect folded N-glycoproteins would be degraded by an ER-associated degradation mechanism (ERAD) after one or multiple rounds of ERQC.

Tunicamycin is an N-glycosylation inhibitor, blocking the formation of N-glycan during the N-glycosylation modification pathway of protein [4]. It was already known that tunicamycin competed with the substrate UDP-GlcNAc and inhibited the synthesis of GlcNAc-PP-Dol by interfering with the GlcNAc phosphotransferase enzyme [2]. N-glycans were essential for the protein folding, stability, transport and function [18]. Additionally, it was also reported that the blocking of protein N-glycosylation modification by tunicamycin resulted in ER stress which caused oxidative stress in cell [2]. So far, N-glycan structures were widely analyzed in mammal’s tissue- and cell-type-specific levels, however, the relative information is very limited in plants, especially in algae [19, 29]. The analysis of N-glycan will not only help unravel the N-linked oligosaccharides structure of proteins, but also provide information for studying the function of the proteins. Therefore, it is interesting to study effects of tunicamycin stress on physiological characteristics and inhibition of synthesis of N-glycan in *P. tricornutum*.

Microalgae being cheap, easy to culture, safe and scalable for accumulating high amount of proteins, therefore, they have emerged as an alternative system for the production of biopharmaceuticals [3, 19]. Several studies have already demonstrated that the diatom *P. tricornutum* is a convenient platform for producing biopharmaceuticals, such as anti-hepatitis B IgG and anti-MARV NP IgG [3]. So far, more than half of the approved biopharmaceutical proteins are N-glycosylated proteins. N-glycosylation is required for recombinant proteins, because the presence and structures of the N-glycans are important for their activity, stability and half-life [19]. It was known that unsuitable or incorrect N-glycosylated proteins will lead to immune reactions, such as fucose epitope on recombinant proteins [23]. Therefore, study of N-glycosylation pathway and N-glycan structures of proteins in biopharmaceutical expression system of *P. tricornutum* is valuable for producing functional and humanized recombinant proteins for clinical therapeutics.

In this study, comprehensive approaches including physiological, transcriptomic, and N-glycomic analyses were used to investigate the N-glycosylation pathway and N-glycan structures in diatom *P. tricornutum* under the tunicamycin stress. The results showed that cell growth, photosynthesis efficiency and soluble protein content were significantly inhibited. Furthermore, the accumulations of soluble sugar and neutral lipid were dramatically enhanced, and the activity of oxidative enzymes and MDA content were remarkably increased under tunicamycin stress. Moreover, the genes related to ERQC and ERAD were differentially expressed in pursuance adapt to the tunicamycin stress. Mannose-type is the major N-glycan structure in *P. tricornutum*, the identification of complex-type N-glycans enriched the database of N-glycan structure in diatom.

## Material and methods

### Microalga and growth conditions

*Phaeodactylum tricornutum* Pt1 (obtained from Oil Crops Research Institute of Chinese Academy of Agricultural Sciences, China). *P. tricornutum* Pt1 cells were initially cultured in f/2 medium at 22℃ under 24 h light condition (50 μmol photons m^−2^ s^−1^). Algal cells were cultured for 5 days to exponential phase in the f/2 medium. Subsequently, transferred to the fresh f/2 medium with 0, 0.1 and 0.3 μg/ml tunicamycin for the next experiments.

### Measurements of physiological parameters

Cell density, *F*v/*F*m, the activity of peroxide dismutase (POD) and superoxide dismutase (SOD), the content of malondialdehyde (MDA) in *P. tricornutum* were measured as described in our previous paper [31]. The soluble protein was analyzed via Coomassie brilliant blue kit (Jiancheng Biotech Company, Nanjing, China) via UV–VIS spectrophotometry at 595 nm [6]. The soluble sugar was analyzed by the methods of [13] using UV–VIS spectrophotometry at 490 nm. The rapid light curve (RLC) was produced using 10 s pulses of actinic light increased from 0 to 1012 (μmol photons m^−2^ s^−1^), then the photosynthetic electron transport (ETR) and the maximal photochemical efficiency of PSII (*F*v/*F*m) was calculated according to pervious study [5]. Cells were collected and stained with Nile red. The fluorescence was measured on black 96-well plate by Multiskan Spectrum (Infinite M200 Pro, Tecan, Switzerland) under the 530 nm excitation and 575 nm emission wavelengths [26].

### Transcriptome analysis

RNA was extracted from algal cells on the 24th hour of tunicamycin stress using the RNAprep pure Plant Kit [Tiangen Biotech (Beijing) Co., Ltd., Beijing, China] and measured using NanoDrop 2000 (Thermo). RNA integrity was analyzed using the RNANano 6000 Assay Kit of the Agilent Bioanalyzer 2100 system (Agilent Technologies, CA, United States). cDNA was synthesized using random primers [cDNA synthesis kit, Tiangen Biotech (Beijing) Co., Ltd.]. NEB Next Ultra TMRNA Library Prep Kit for Illumina (NEB, United States) was used to generate sequence libraries. Sequence reads are available on the NCBI sequence read archives [GSE209809].

The library construction of transcriptomic and bioinformatic analysis were conducted as described in our previous paper [31]. Genes with |fold change|≥ 1.5 and FDR < 0.01 (adjusted P-value, determined by the Benjamini and Hochberg multiple-testing correction implemented in the ‘p. adjust’ method of R) were defined as differentially expressed genes. The value of FPKM was the average of three biological replicates.

### N-glycoprotein and N-glycan structure analysis

Algal cells were collected on the 24th hour of tunicamycin stress. 1.5 mg fresh algal cells were grinded by liquid nitrogen into cell powder and used for the protein extraction described in our previous paper [29]. The concentration of protein was measured via BCA kit according to the instructions of manufacturer (ab102536, Abcam, China). Subsequently, the protein was digested by trypsin overnight at 1:50 (trypsin: protein) mass ratio and 4 h at 1:100 (trypsin: protein) mass ratio [10]. The tryptic peptides were fractionated into 60 fractions by high pH reverse-phase high performance liquid chromatography using Thermo BetaSil C18 column [11]. The fractionated N-glycopeptides were first enriched by hydrophilic interaction liquid chromatography (HILIC) microcolumn, collected by 10% acetonitrile and dried by vacuum freezing [28]. After drying, the glycopeptides were digested in PNGase A and F at 37 ℃ overnight. The digested glycopeptides were washed by C18 ZipTips and lyophilized for liquid chromatography tandem mass spectrometry (LC–MS/MS) analysis. The procedure and data setting were carried out according to method stated by [21], and [29] Compared to that, in the wild type, N-glycopeptides with ratio ≥ 1.5 and p value < 0.05 were defined as differentially expressed N-glycopeptides.

### Data analysis

Data from three replicate experiments were shown as mean ± standard deviation (SD). SPSS software (version 25.0) with one-way ANOVA followed by comparison method (LSD test) were used to analyze the data. * represents significant differences in mean value among different treatments (*P* < 0.05). ** represents extremely significant differences in mean value among different treatments (*P* < 0.01). Origin 8.5 and Adobe Illustrator 2020 were used to create figures.

## Results

### Effect of tunicamycin on the physiological characteristics of *P. tricornutum*

**Fig. 1:**
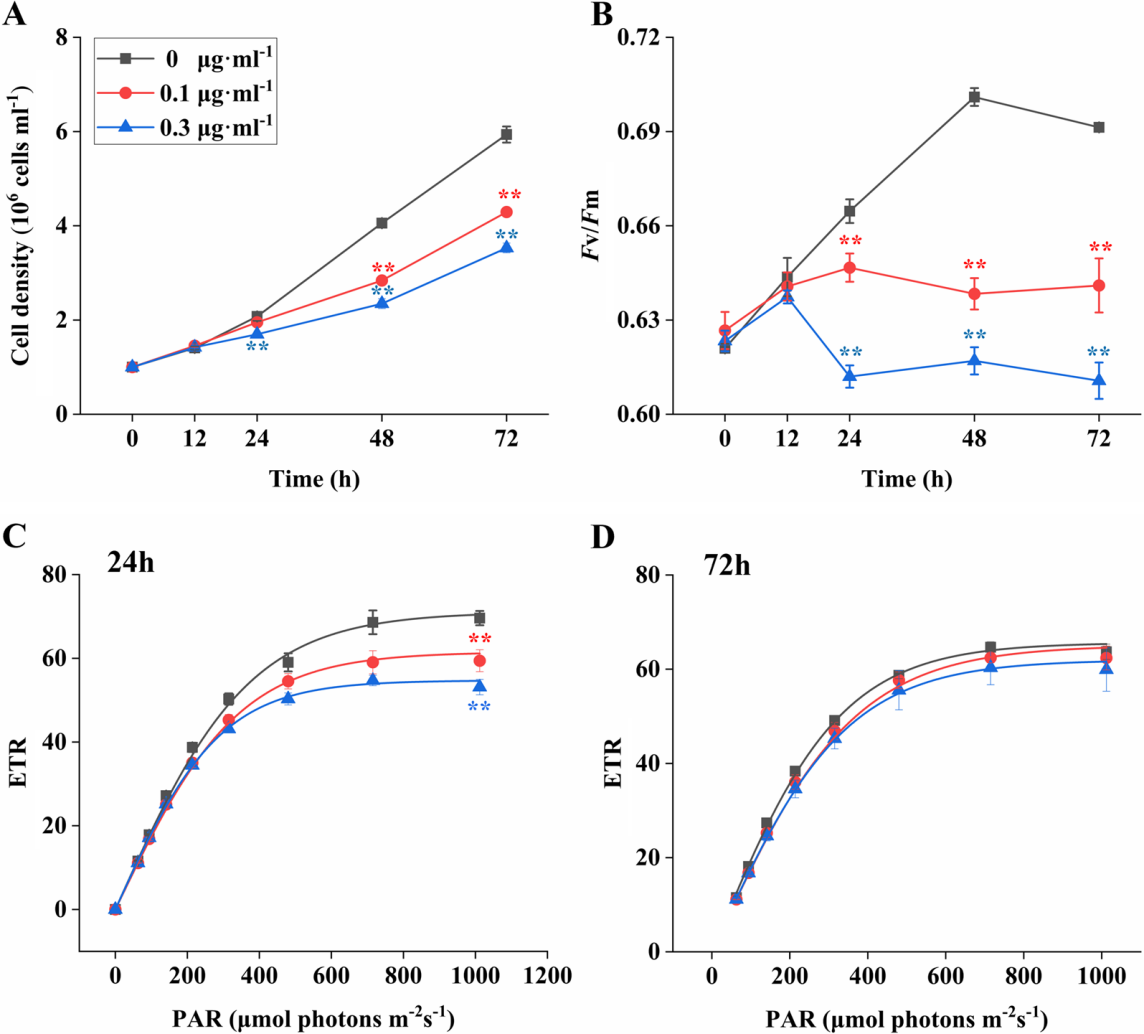
Cell density, *F*v/*F*m and ETR of *P. tricornutum* under the tunicamycin stress. *ETR* the rate of photosynthetic electron transport, *PAR* light intensity

It was shown that the cell density of *P. tricornutum* was extremely significantly decreased on the 48th hour of 0.1 μg/ml tunicamycin stress and on the 24th hour of 0.3 μg/ml tunicamycin stress (P < 0.01, Fig. 1A). Algal *F*v/*F*m was statistically remarkably inhibited on the 24th hour of tunicamycin stresses (P < 0.01, Fig. 1B). Meanwhile, the rate of photosynthetic electron transport (ETR) was measured, the results were shown in Fig. 1C and D. It was observed that ETR, tunicamycin treated groups, were significantly decreased on the 24th hour as compared to the control group.

**Fig. 2:**
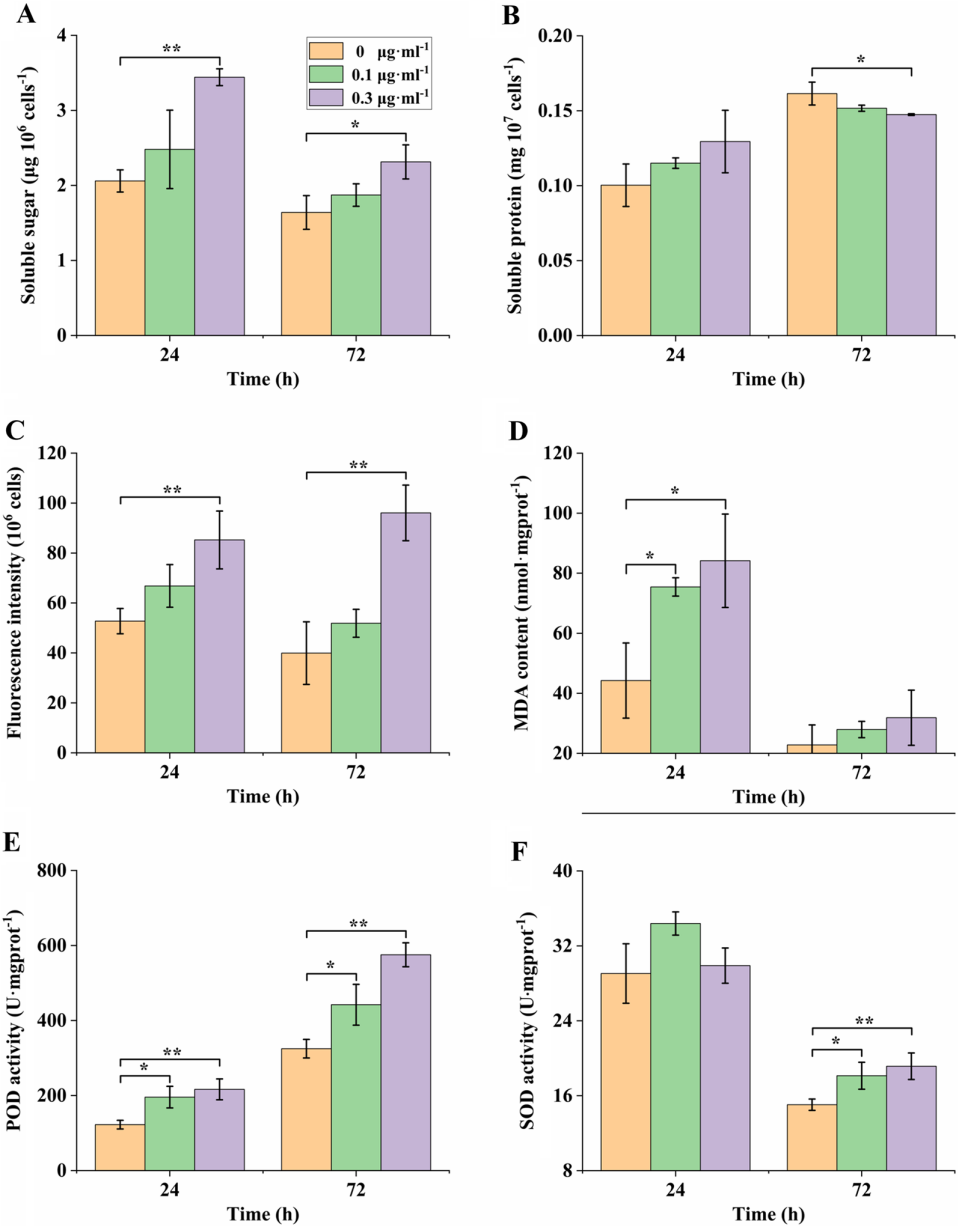
Soluble sugar and protein, lipid, MDA, POD and SOD of *P. tricornutum* under the tunicamycin stress. *MDA* malondialdehyde, *POD* peroxide dismutase, *SOD* superoxide dismutase.

In addition to the cell density and photosynthesis, soluble sugar, protein, lipid, and the activity of anti-oxidases were analyzed, as shown in Fig. 2. It was found that the accumulation of sugar was significantly stimulated under the high concentration of tunicamycin stress (P < 0.05, Fig. 2A). However, the accumulation of soluble protein was inhibited, especially on the 72nd hour under the 0.3 μg ml^−1^ tunicamycin (Fig. 2B). The accumulation of neutral lipid content was similar with that of sugar, remarkably increasing under the 0.3 μg ml^−1^ tunicamycin (Fig. 2C). The analysis of MDA, POD and SOD showed that the MDA content was significantly increased on the 24th hour of tunicamycin stresses (P < 0.05, Fig. 2D), the activity of POD was markedly enhanced on the 24th and 72nd hours stress (P < 0.05, Fig. 2E), while the SOD activity was only signally strengthened on the 72nd hour stress (P < 0.05, Fig. 2F).

### Effect of tunicamycin on the gene expression related to ERQC and ERAD

Subsequently, the expression of genes under the 0.3 μg ml^−1^ tunicamycin stress on the 24th hour was analyzed via transcriptome. Among all genes involved in the ERQC pathway, the expression of 18 genes were up-regulated, and only 1 gene was down-regulated (Table 1, Additional file 2: Table S1). ALG13, ALG2, ALG3, ALG12, ALG8, STT3, GSII, CRT, PDI, NEF, HSP, Bip and GRP were involved in the ER N-glycosylation pathway of proteins. GSII, CRT, PDI, NEF, HSP, Bip and GRP genes participated in the key pathway of ERQC. While GnTI, FucT and XylT were involved in the Golgi pathway of ERQC for the mature of N-glycan structures.

**Table 1:** The expression of genes involved in ERQC pathway

Gene ID	Abbreviation	Domain	Log_2_ FC	padj
9427	ALG 13	PF04101	1.67E + 00	1.83E − 06
22,554	ALG 2	PF00534	1.31E + 00	5.96E − 05
10,976	ALG 3	PF05208	6.52E − 01	3.20E − 04
44,425	ALG 12	PF03901	1.14E + 00	3.52E − 05
44,905	ALG 8	PF03155	1.10E + 00	1.10E − 03
55,198	STT3	PF02516	6.22E − 01	2.36E − 04
54,169	GSII	PF07915	5.96E − 01	4.92E − 02
41,172	CRT	PF00262	1.23E + 00	1.57E − 07
54,844	GnT I	PF03071	1.79E + 00	3.57E − 05
46,110	FucT	PF00852	2.25E + 00	5.55E − 11
45,496	XylT	PF04577	1.74E + 00	9.20E − 11
2808	PDI1	PF00085	7.79E − 01	5.45E − 03
42,566	PDI2	PF00085	8.02E − 01	1.75E − 05
2242	NEF1	PF01369 PF12782	− 1.06E + 00	1.11E − 07
2623	NEF2	PF01369 PF12783	5.85E − 01	1.76E − 02
37,590	HSP70G1	PF05180	1.49E + 00	1.39E − 06
46,333	HSP70G2	PF00012	7.77E − 01	2.36E − 03
21,519	Bip1	PF00012	1.31E + 00	1.04E − 13
2643	Bip2	PF00012	1.05E + 00	1.40E − 03
16,786	GRP94	PF00183 PF02518	1.72E + 00	7.67E − 10

Log_2_FC > 0 represent upregulation, whereas Log_2_FC < 0 represent downregulation under tunicamycin stress. All presented fold changes are statistically significant, q-value < 0.05

ALG13, β-(1,4)-N-acetylglucosaminyltransferase 13; ALG2/3, α-(1,3)-mannosyltransferase 2/3; ALG12, α-(1,2)- mannosyltransferase 12; ALG8, α-(1,3)-glucosyltransferase 8; OST, oligosaccharyltransferase; STT3, a subunit of OST; GSII, α-glucosidase II; CRT, calreticulin; GnT I, β-N-acetylglucosaminyltransferase; FucT, α-(1,3)-fucosyltransferase; XylT, β-(1,2)-xylosyltransferase; PDI, protein disulfide isomerase; NEF, nucleotide exchange factor; HSP70, heat shock protein 70; Bip, binding protein; GRP94, glucose regulating protein 94.

After the quality control, incorrect and unfolded proteins will be exported from the ERQC pathway and then degraded via ERAD mechanism. Therefore, in addition to ERQC pathway, ERAD pathway was also analyzed. Genes involved in different steps of ERAD were analyzed, including genes in the recruitment of ERAD substrates, ubiquitination of chosen ERAD clients, retro-translocation of ERAD substrates, substrate extraction, processing, and delivery to the proteasome. Among all genes related with ERAD mechanism, 30 genes were differentially expressed, including 18 down-regulated and 12 up-regulated genes (Table 2, Additional file 3: Table S2). These differentially expressed genes included Doa, Uba, Ubc, Ube, RAD, Npl, Ufd, Png, Ubx, cdc48 and Otu. Genes related with recruitment and retro-translocation of ERAD substrates were not differentially expressed. However, three Doa, one Uba, eight Ubc and six Ube genes related with ubiquitination of chosen ERAD proteins were differentially expressed. One RAD, Npl, Ufd, Png, Ubx, cdc48 and Otu related with substrate extraction, processing, and delivery to the proteasome were also differentially expressed.

**Table 2:** The expression of genes involved in ERAD mechanism

Gene ID	Abbreviation	Domain	Log_2_ FC	padj
12,887	Doa1	PF12906	− 9.95E − 01	1.09E − 08
42,799	Doa2	PF12906	5.99E − 01	2.98E − 04
33,774	Uba3	PF08825	9.55E − 01	6.05E − 06
30,389	Ubc1	PF00627 PF00179﻿	− 8.23E − 01	2.38E − 08
22,525	Ubc2	PF00179	− 1.39E + 00	1.01E − 13
20,308	Ubc 1C	PF00179	− 7.24E − 01	6.81E − 04
30,092	Ubc 5A	PF00179	− 1.32E + 00	1.65E − 03
6645	Ubc6	PF00179	9.77E − 01	2.44E − 02
37,077	Ubc J2	PF00179	− 7.39E − 01	2.74E − 02
48,512	Ubc Q2	PF00179	− 6.14E − 01	3.04E − 03
10,724	Ubc W-B	PF00179	1.24E + 00	9.65E − 06
2541	Ube1	PF00632	− 8.90E − 01	1.67E − 04
52,750	Ube2	PF00632 PF00627	− 7.44E − 01	8.82E − 04
14,177	Ube3	PF00632	− 1.53E + 00	8.46E − 03
383	Ube4	PF00632	− 1.35E + 00	2.12E − 02
14,345	Ube5	PF00632	− 1.04E + 00	2.90E − 02
44,885	Ube6	PF00632	8.37E − 01	3.87E − 02
47,158	Ubx1	PF00789	− 2.20E + 00	2.05E − 08
44,122	Ubx2	PF00789	6.69E − 01	1.18E − 03
49,430	Ufd1	PF03152	− 1.05E + 00	6.83E − 04
40,273	Rad23	PF00627	− 1.69E + 00	6.45E − 05
45,135	Npl4	PF05021	9.23E − 01	4.41E − 07
7656	Png1	PF00085 PF09409	2.11E + 00	1.22E − 07
41,252	Otu2	PF02338	9.88E − 01	1.24E − 03
36,597	Scdc48	PF02359 PF00004 PF17862	7.58E − 01	1.61E − 03

Log_2_FC > 0 represent upregulation, whereas Log_2_FC < 0 represent downregulation under tunicamycin stress. All presented fold changes are statistically significant, q-value < 0.05

Doa, putative degradation of alpha-2 protein; Ubc, ubiquitin-conjugating enzyme E2; Ube, ubiquitin-protein ligase E3; Ubx, ubiquitin regulatory X domain-containing protein; Ufd, ubiquitin fusion degradation protein; Upl, ubiquitin-protein ligase; Rad 23, ubiquitin receptor Rad 23; Npl, substrate-recruiting factor Npl; Png, peptide: N-glycanase; Otu, ubiquitin thioesterase Otu; Cdc, cell division control protein 48.

### Effect of tunicamycin on the N-glycan structure

**Fig. 3:**
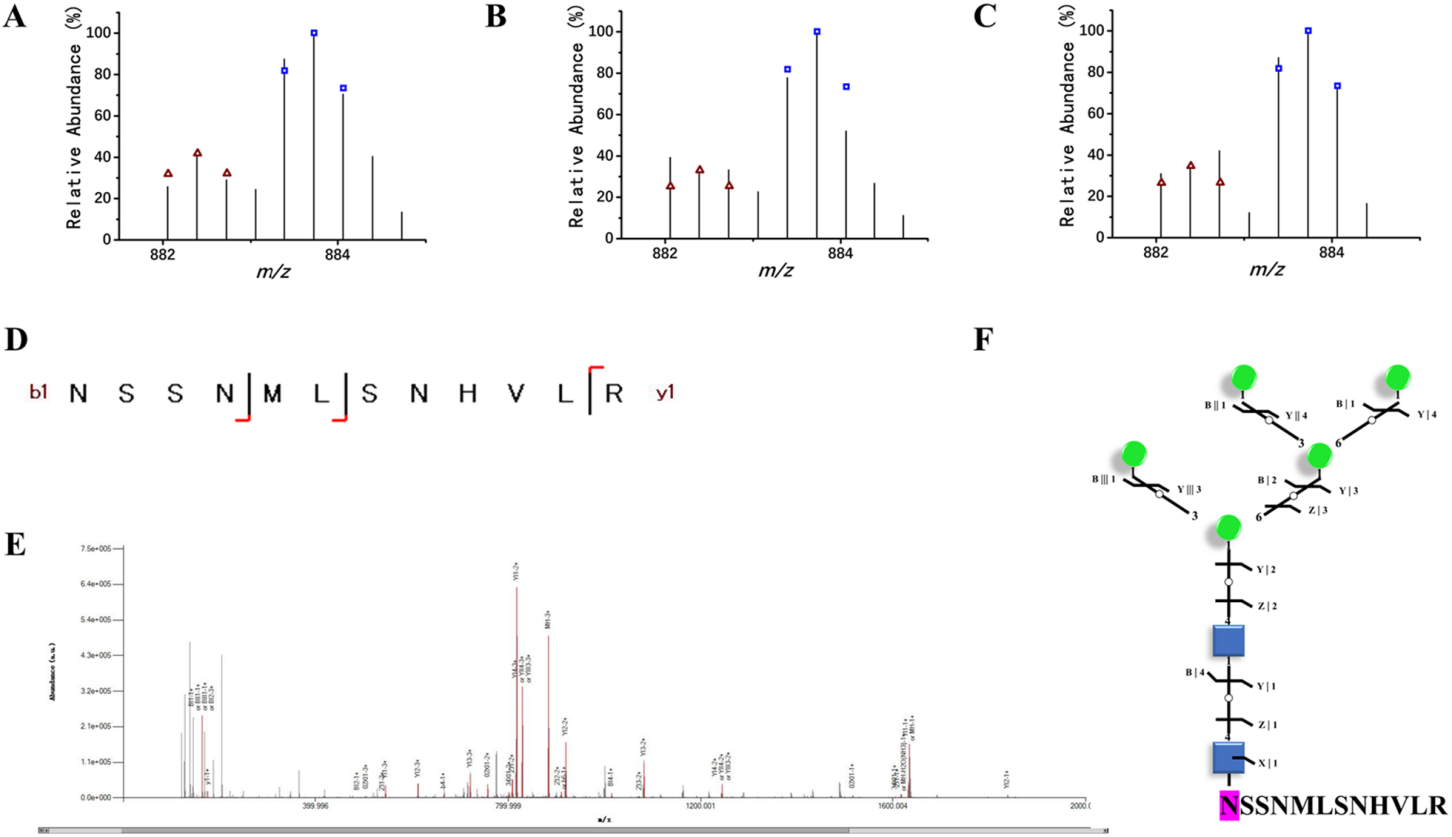
Up-regulated intact N-glycopeptide NSSNMLSNHVLR. **A**–**C** Isotopic envelope fingerprinting maps of the paired precursor ions from the three technical replicates; **D** Graphical fragmentation map of the peptide backbone; **E** Annotated MS/MS spectrum with the matched fragment ions marked; **F** Graphical fragmentation map of the N-glycan moiety. Green circle, mannose; blue square, N-acetylglucosamine

**Fig. 4:**
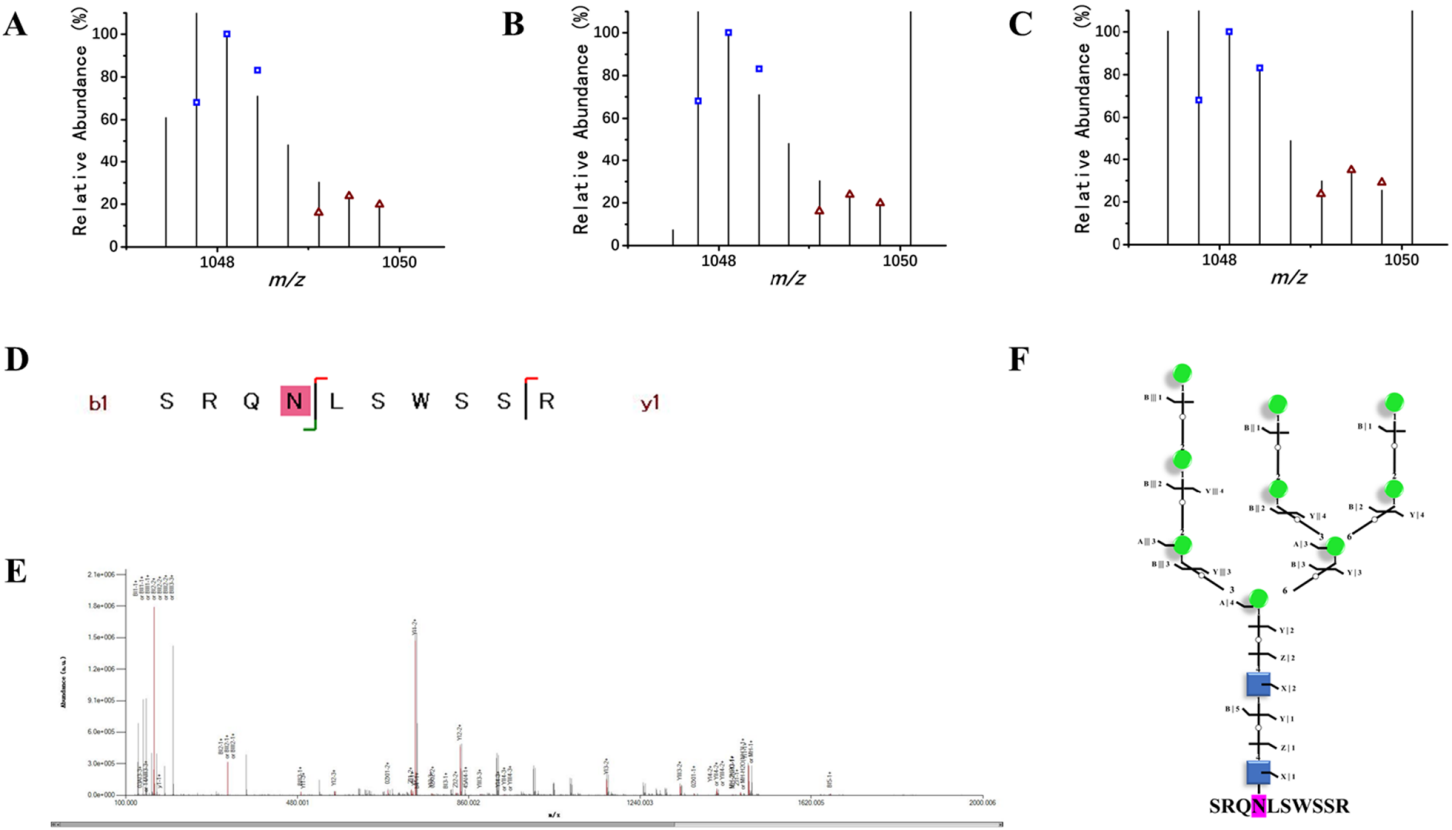
Down-regulated intact N-glycopeptide SRQNLSWSSR. **A**–**C** Isotopic envelope fingerprinting maps of the paired precursor ions from the three technical replicates; **D** Graphical fragmentation map of the peptide backbone; **E** Annotated MS/MS spectrum with the matched fragment ions marked; **F** Graphical fragmentation map of the N-glycan moiety. Green circle, mannose; blue square, N-acetylglucosamine

To further clarify the effects of tunicamycin on the N-glycan structure, N-glycomic analysis was performed. Total, 502 N-glycoproteins corresponding to 556 N-glycosites, 1021 intact N-glycopeptides were obtained and analyzed in this study. Among them, 110 N-glycopeptides were differentially expressed, including 78 up-regulated and 32 down-regulated N-glycopeptides (Additional file 2 and Additional file 3: Tables). It was shown that the N-glycans from 78 up-regulated N-glycopeptides had 3 to 9 mannose residues, belonging to mannose-type structure. However, among the 32 down-regulated N-glycopeptides, 5 N-glycans were complex-type N-glycan structure, 27 were mannose-type structure with 5–9 mannose residues. Besides, the N-glycan structure of ERQC related proteins, such as alpha-mannosidase modified by one N-glycan structure (Glc1GlcNAc2Man9), xylosyltransferase modified by three N-glycan structures (GlcNAc2Man9, Glc1GlcNAc2Man9 and Fuc1Xyl1GlcNAc2Man7), mannosyltransferase modified by two structures (GlcNAc2Man9 and Glc1GlcNAc2Man9), beta-glucosidase modified by GlcNAc2Man9 structure, and ERAD related protein, E3 ubiquitin transferase modified by GlcNAc2Man9 structure were identified from this study. Compared to wild type, the expression of protein B7G766 with N-glycan structure (GlcNAc2Man5) was upregulated (Fig. 3). While the expression of protein B7GD12 with N-glycan structure (GlcNAc2Man9) was downregulated (Fig. 4). In addition to these two N-glycans, a mannose-type and complex-type N-glycans were shown in Additional file 1: Fig. S1.

## Discussions

Tunicamycin is a well-known ER stress inducer, because it inhibits the transfer of GlcNAc-1-phosphate to dolichol monophosphate, restraining the first step of the protein N-glycosylation modification [2]. Therefore, the effects of tunicamycin on the physiological, gene expression and N-glycan structure were studied here in diatom *P. tricornutum*. The cell density of *P. tricornutum* was significantly reduced by tunicamycin in a dose-dependent manner. Similarly, the mortality rate of plants and the cell viability of human endothelial cells were also dependent on the concentration of tunicamycin [25, 27]. The efficiency of photosynthesis (*F*v/*F*m) and the rate of photosynthetic electron transport (ETR) were significantly inhibited by tunicamycin stress, indicating that tunicamycin might result in the differentially expression of genes related to photosynthesis. The significant difference of ETR was observed on the 24th hour but not on the 72nd hour, this phenomenon might be explained by a recovery on the 72nd hour of tunicamycin stress. According to the transcriptomic analysis, it was found that some genes related to chlorophyll were differentially expressed, such as the downregulation of chlorophyll A-B binding protein genes (Phatr3_J17766, Phatr3_J11006, Phatr3_J30648, Phatr3_J23257, Phatr3_J30643 and Phatr3_J50705) and cytochrome c gene (Phatr3_J44056), and the upregulation of Phatr3_J32294 and Phatr3_J42519. It was reported that the downregulation of chlorophyll A-B binding protein gene effected the photosynthesis in *Chlorella sorokiniana* [12]. Therefore, these differentially expressed genes might be related with the inhibition of photosynthesis.

Genes with red mark were up-regulated, while genes with green mark were down-regulated under the tunicamycin stress. Black marked genes were not differentially expressed in this study. Green circle, mannose; blue square, N-acetylglucosamine; red triangle, glucose; yellow star, xylose; purple square, fucose.

**Fig. 5:**
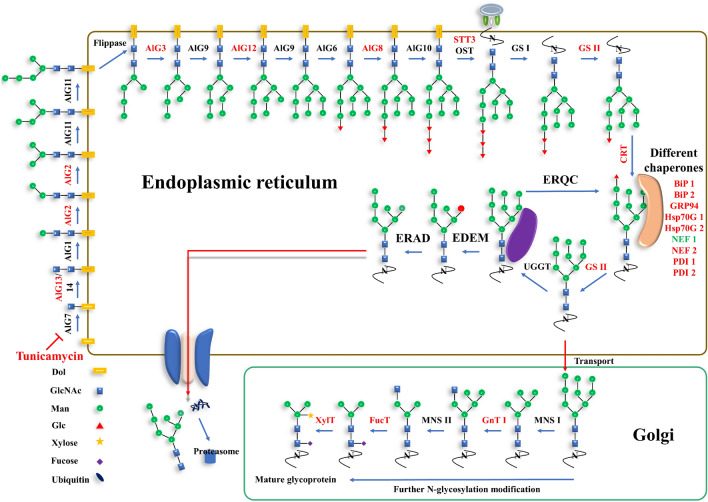
The working model of ERQC pathway in *P. tricornutum*

Under the stress of tunicamycin, the protein N-glycosylation and homeostasis would be interrupted, resulting in the decrease of total soluble protein. It was well known that carbon and nitrogen metabolisms are to some extent balance in cell. Therefore, it was observed that soluble sugar and neutral lipid were remarkably increased, while soluble protein was dramatically decreased under the high dose of tunicamycin. Previous study reported that more than half of secretory and membrane proteins were N-glycosylated proteins [16]. Therefore, the decreased soluble protein might be related with differentially expressed genes during the ERQC pathway and ERAD mechanism. An accurate and efficient protein N-glycosylated modification and ERQC pathway are necessary for the protein quality control and its content. Based on previous papers and bioinformatic analysis in *silico*, the working model of ERQC pathway was proposed (Fig. 5) [1, 6, 14]. After the inhibition of tunicamycin on the first step of protein N-glycosylation modification, the expression of ALG genes, STT3 subunit of OST, CRT and PDI genes were upregulated to orchestrate the protein N-glycosylation modification. CRT and PDI are the key chaperones in the ERQC to recognize and combine the new synthesized N-glycoprotein for quality control (Nagashima et al. 2018b). The upregulation of CRT and PDI might be important for regulating the unfolded and/or incorrect N-glycoproteins under the stress of tunicamycin. STT3 played an important role in the protein N-glycosylation efficiency in plants [9], suggesting that the upregulation of STT3 gene expression in *P. tricornutum* might enhance the N-glycosylation modification of protein under the tunicamycin stress. In addition to the genes in ER, the expression of GnTI, FucT and XylT genes in Golgi apparatus were also upregulated. These genes are important for the maturation of N-glycans in *P. tricornutum*. It was known that GnTI was responsible for the formation of complex-type N-glycan structure in *P. tricornutum* [1]. While FucT (ID:54,599) was found for the alpha-1,4-fucose modification of N-glycan in *P. tricornutum* in our recent study (unpublished).

Due to the differentially expressed genes during the pathway of protein N-glycosylation, it is interesting to know the N-glycan structure under tunicamycin stress. So far, the N-glycan structures were widely identified in animals and higher plants, however, the relative information in microalgae is very limit [6]. The N-glycan structures in diatom *P. tricornutum* were previously reported to be mannose-type ranging from Man-5 to Man-9 via MALDI-TOF MS, IMS-MS and ESI-MS^n^ fragmentation patterns data [1, 7, 17]. Subsequently, addition to the mannose-type, a hybrid N-glycan with a terminal GlcNAc residue was identified in *P. tricornutum* via C18-RPLC-MS/MS (HCD) analysis [29]. In this study, mannose-type with 3 to 9 mannose residues was identified as the primarily N-glycan structure through the high throughput method. Although complex N-glycans were also speculated in *P. tricornutum* [30], complex N-glycans with fucose and xylose residues were qualit in this study via C18-RPLC-MS/MS (HCD) analysis. Therefore, this study updated the existing N-glycan structure database of *P. tricornutum*. The global profiling of N-glycans here provide important information for further studying the function of N-glycosylation modification on proteins. Consistent with the increased content of MDA, enhanced activity of POD and SOD, the differentially N-glycosylation modifications might change the functions of N-glycoproteins and then played a role in alleviating the tunicamycin stress. The N-glycan structures of ERQC and ERAD related proteins were also identified here, however, the function of these N-glycan structures on the proteins is still unknown and needs more investigations to clarify.

**Fig. 6:**
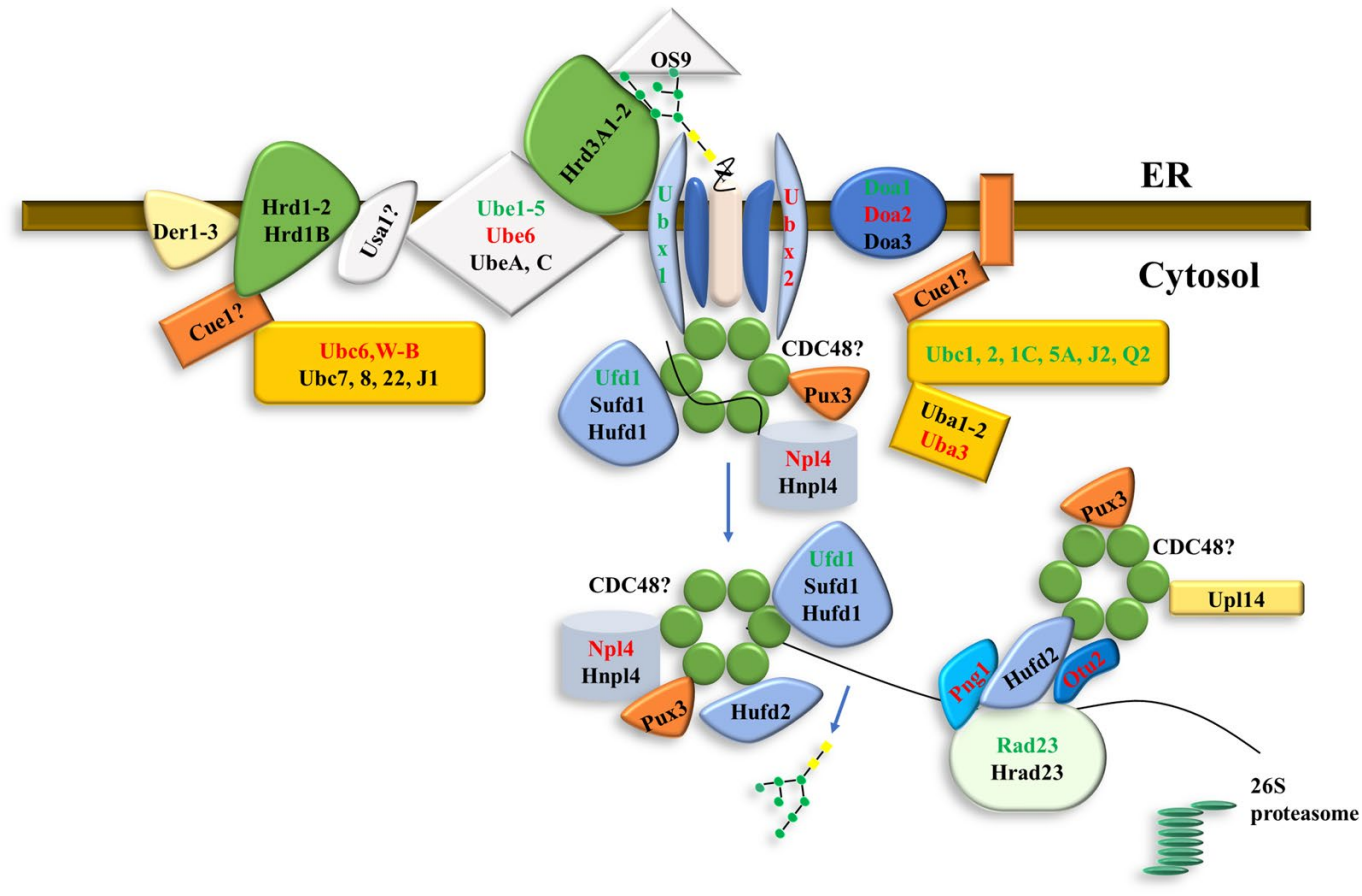
The working model of ERAD mechanism in *P. tricornutum* under tunicamycin stress. Genes with red marks were up-regulated, while genes with green marks were down-regulated under the tunicamycin stress. Black marked genes were not differentially expressed in this study.

After the ERQC pathway, incorrect or unfolded proteins will be degraded by ERAD mechanism [15]. The working model was speculated in *P. tricornutum* under the tunicamycin stress, as shown in Fig. 6. During the ERAD mechanism, the recruitment of ERAD substrates is the first step. Incomplete/misfold proteins with alpha-1,6-Man residue N-glycans will be recognized and bind by OS9 and Hrd3A [6]. However, the related genes were not differentially expressed under the tunicamycin stress. After the recruitment of ERAD substrates, the chosen ERAD proteins would be ubiquitinated by a series of enzymes. During this step, some differentially expressed genes might be responsible for the ubiquitination of incorrect and/or unfolded proteins. Hrd1 complex was responsible for the ubiquitination of ER luminal and membrane proteins, while Doa2 for the cytosolic proteins [15]. In this study, ubiquitination reaction was speculated to be activated by Uba3, conjugated by Ubc6/W/B, ligated by Ube1-6 [22]. Genes related to retro-translocation of ERAD substrates (e.g. Hrd genes) were not differentially expressed under the tunicamycin stress. However, Hrd genes such as Hrd3A1, Hrd3A2, Hrd1 and Hrd1B were significantly differentially expressed under the nitrogen limitation in *P. tricornutum* [24]. Therefore, it was speculated the expression of genes related with retro-translocation of ERAD substrates might dependent on different stresses. A homohexameric Cdc48-Ufd1-Npl4 complex was required for the substrate extraction. This complex was recruited to the Hrd1/Doa2 E3 complex by Ubx2. The up-regulated Ubx2 was proposed for the recruitment of the Cdc48-Ufd1-Npl4 complex in this study. Subsequently, ERAD proteins were deubiquitinated by enzyme Otu2, de-glycosylated by the Png1 and delivered to the cytosolic proteasome via Cdc48-Rad23-Dsk complex for the final degradation [6]. The whole working model explained the decreased protein content under the tunicamycin stress.

## Supplementary Information

**Additional file 1: Figure S1.** Up-regulated intact N-glycopeptide ALNSSNTER. **Figure S2**. Up-regulated intact N-glycopeptide DGVATNVCPR.

**Additional file 2: Table S1.** Differentially expressed genes related with ERQC and ERAD.

**Additional file 3: Table S2.** The up- and down-regulated N-glycopeptides.
