# Supplementary material for: Endoplasmic reticulum-quality control pathway and endoplasmic reticulum-associated degradation mechanism regulate the N-glycoproteins and N-glycan structures in the diatom Phaeodactylum tricornutum
Source: Microb Cell Fact. 2022 Oct 20;21:219. doi: 10.1186/s12934-022-01941-y (PMC9585838; doi:10.1186/s12934-022-01941-y)
Supplement: Supplementary file 1 — Additional file 1: Figure S1. Up-regulated intact N-glycopeptide ALNSSNTER. Figure S2. Up-regulated intact N-glycopeptide DGVATNVCPR. [file 12934_2022_1941_MOESM1_ESM.docx]

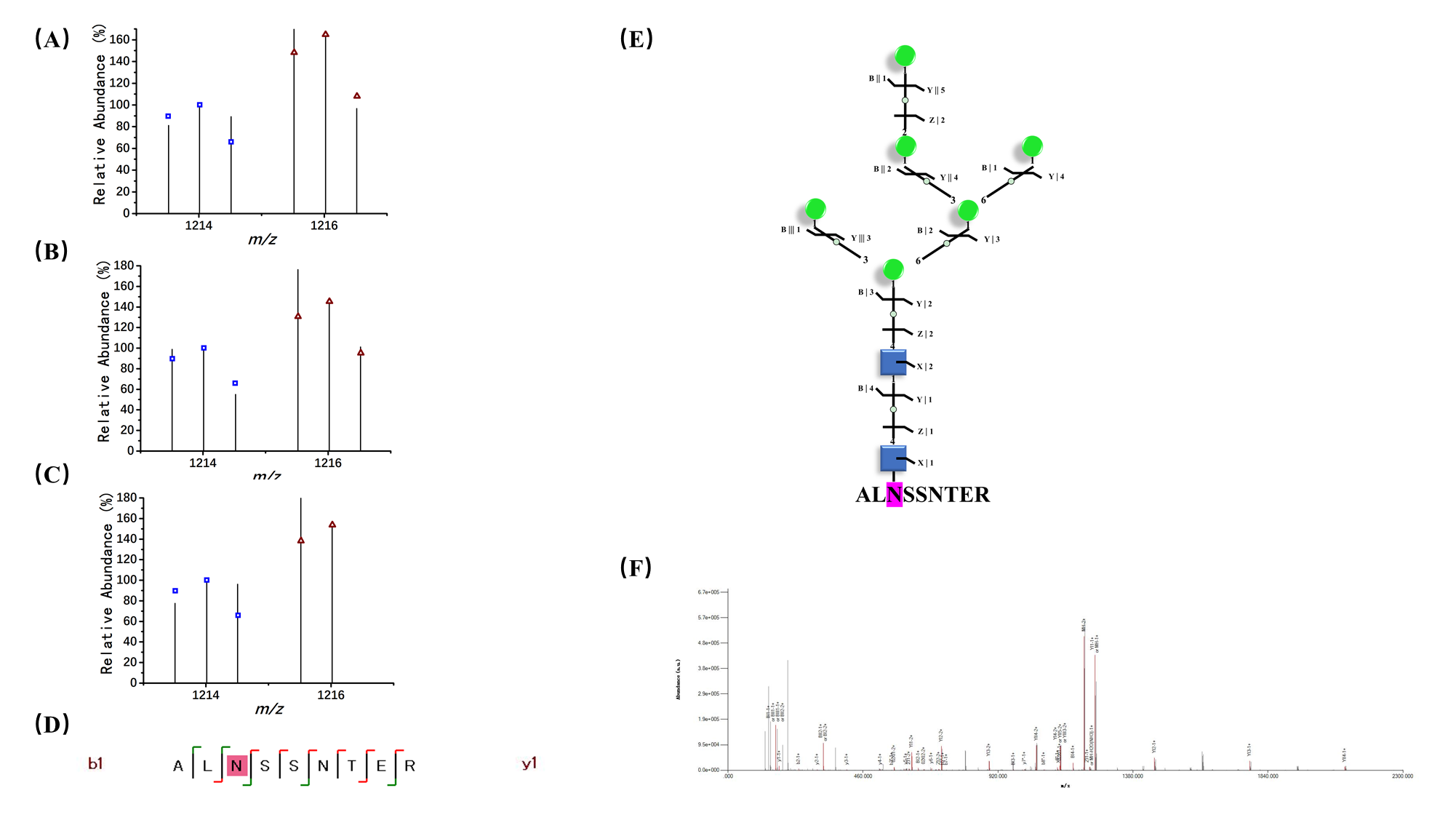


Figure S1 Up-regulated intact N-glycopeptide ALNSSNTER. (A-C) Isotopic envelope fingerprinting maps of the paired precursor ions from the three technical replicates; (D) Graphical fragmentation map of the peptide backbone; (E) Graphical fragmentation map of the N-glycan moiety; (F) Annotated MS/MS spectrum with the matched fragment ions marked. Green circle, mannose; blue square, N-acetylglucosamine.


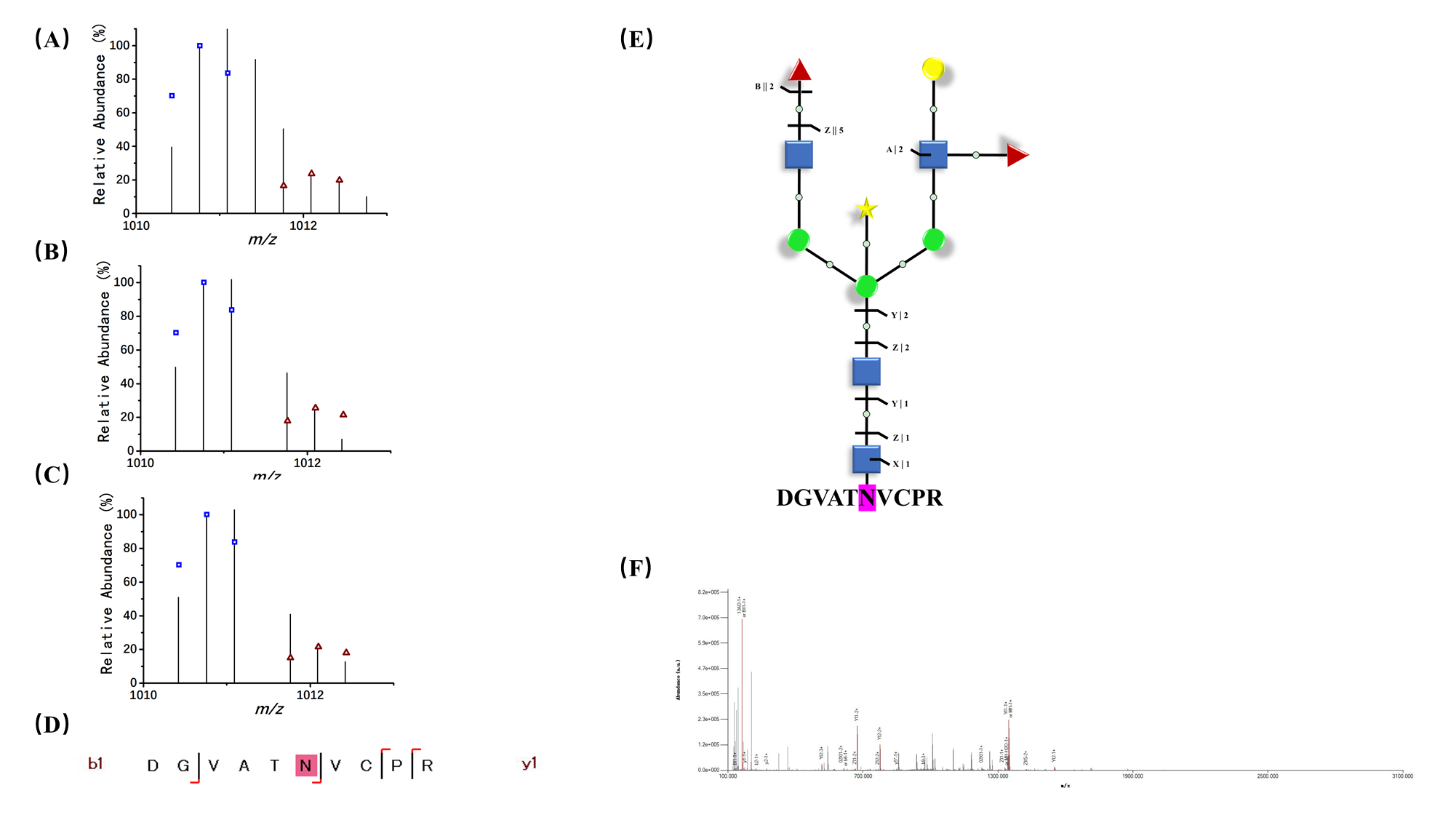


Figure S2 Up-regulated intact N-glycopeptide DGVATNVCPR. (A-C) Isotopic envelope fingerprinting maps of the paired precursor ions from the three technical replicates; (D) Graphical fragmentation map of the peptide backbone; (E) Graphical fragmentation map of the N-glycan moiety; (F) Annotated MS/MS spectrum with the matched fragment ions marked. Green circle, mannose; blue square, N-acetylglucosamine; red triangle, fucose; yellow star, xylose; yellow circle, galactose.
